# Neural plasticity and migraine

**DOI:** 10.1186/1129-2377-16-S1-A26

**Published:** 2015-09-28

**Authors:** Gianluca Coppola

**Affiliations:** Department of Neurophysiology of Vision and Neurophthalmology, G.B. Bietti Foundation IRCCS, Rome, Italy

## Background

During recent years, various experimental data suggested that the functional state of the migraineur's brain fluctuates in relation with the cyclical recurrence of the migraine attack. This was historically observed with the methods of clinical neurophysiology that revealed interictal deficient habituation of any kind of sensory responses - with the notable exception of the olfaction - that was attributed to abnormal thalamo-cortical interactions and its normalization during an attack. On the other hand, studies with repetitive transcranial magnetic stimulation (rTMS), have reported interictal paradoxical cortical responses in reaction to both depressing or enhancing rTMS and their changes up to the bending point of an attack when cortical responsivity behaves differently.

Such recurring changes were confirmed recently with morphological and functional neuroimaging methodologies. For instance, fMRI BOLD responses induced by painful stimuli differed between ictal and interictal scans in episodic migraine. We recently showed that cyclic changes can also be demonstrated at rest, i.e. without any sensory input, in anisotropy of thalamic microstructure, in grey matter density of temporo-parietal areas, and in interconnectivity among large scale cortical networks. The conjunction of neuroimaging and neurophysiological data can be considered as robust evidence favouring cycling morphological and functional brain alterations as prominent features of migraine pathophysiology.

## Conclusions

Both the abnormal neurophysiological information processing and morphological brain changes point to altered neural plasticity mechanisms, which prevent the immediate and longer-lasting cortical changes that allow multimodal sensory integration and reflect adaptation to headache recurrence.

